# A New Peritoneal Dialysis Solution Containing L-Carnitine and Xylitol for Patients on Continuous Ambulatory Peritoneal Dialysis: First Clinical Experience

**DOI:** 10.3390/toxins13030174

**Published:** 2021-02-24

**Authors:** Carmela Rago, Teresa Lombardi, Giorgia Di Fulvio, Lorenzo Di Liberato, Arduino Arduini, José C. Divino-Filho, Mario Bonomini

**Affiliations:** 1 Nephrology and Dialysis Unit, Department of Medicine, G. D’Annunzio University of Chieti-Pescara, SS. Annunziata Hospital, Via dei Vestini, 66013 Chieti, Italy; carmeenrago@gmail.com (C.R.); terelomb@hotmail.it (T.L.); difulvio.giorgia@gmail.com (G.D.F.); lorenzo.diliberato@asl2abruzzo.it (L.D.L.); 2 Department of Research and Development, Iperboreal Pharma, 65100 Pescara, Italy; a.arduini@iperboreal.com; 3 Division of Renal Medicine, CLINTEC, Karolinska Institutet, 171 77 Stockholm, Sweden; jose.divino@ki.se

**Keywords:** end-stage renal disease, peritoneum, peritoneal dialysis, CAPD, carnitine, xylitol, PD fluid, solution

## Abstract

Peritoneal dialysis (PD) is a feasible and effective renal replacement therapy (RRT) thanks to the dialytic properties of the peritoneal membrane (PM). Preservation of PM integrity and transport function is the key to the success of PD therapy, particularly in the long term, since the prolonged exposure to unphysiological hypertonic glucose-based PD solutions in current use is detrimental to the PM, with progressive loss of peritoneal ultrafiltration capacity causing technique failure. Moreover, absorbing too much glucose intraperitoneally from the dialysate may give rise to a number of systemic metabolic effects. Here we report the preliminary results of the first clinical experience based on the use in continuous ambulatory PD (CAPD) patients of novel PD solutions obtained through partly replacing the glucose load with other osmotically active metabolites, such as L-carnitine and xylitol. Ten CAPD patients were treated for four weeks with the new solutions. There was good tolerance to the experimental PD solutions, and no adverse safety signals were observed. Parameters of dialysis efficiency including creatinine clearance and urea Kt/V proved to be stable as well as fluid status, diuresis, and total peritoneal ultrafiltration. The promising tolerance and local/systemic advantages of using L-carnitine and xylitol in the PD solution merit further research.

## 1. Introduction

There is an increasing worldwide number of end-stage renal disease (ESRD) patients requiring chronic renal replacement therapy (RRT), which represents a significant economic burden on any health system [1]. Peritoneal dialysis (PD) is a consolidated, cost-effective, home care form of RRT suitable for ESRD, which exploits as its biological dialysis membrane the semipermeable peritoneum [2]. As compared with hemodialysis (the most commonly used dialysis modality), PD is less expensive, has a similar survival rate, preserves residual kidney function better, removes fluid and solutes more gradually and continuously, and cuts right down on cardiac stress [3].

In PD, removal of excess water and retained uremic solutes from the patient’s blood (dialytic exchange) occurs through the introduction into the peritoneal cavity, via an implanted intra-abdominal catheter, of a PD solution (also called PD fluid; dialysate). According to the three-pore model, which well describes the peritoneal membrane (PM) function, the capillary endothelium is the membrane’s main transport barrier [4]. The PD solution has a usual volume of two liters and contains electrolytes (sodium, magnesium, calcium, chloride), a buffer (lactate and/or bicarbonate), and an osmotic agent in order to remove the daily excess fluid from the patient (peritoneal ultrafiltration, UF) and to enhance convective transport (peritoneal clearance). Following a 4–8 h dwell time, the effluent is drained and fresh dialysate reinfused. One may perform this manually (continuous ambulatory PD; CAPD) via 4–5 daily exchanges, or use an automated cycler (automated PD; APD), usually during the night (a process lasting 8–10 h).

Although PD is a viable treatment for ESRD, it is prescribed in only a minority of dialysis patients [3,5]. The explanation for such a discrepancy lies mainly in certain major limitations concerning PD efficiency and sustainability [6]. In fact, bioincompatibility of the dialysis fluid forms the principal problem for long-term PD patients, since the anatomical and functional integrity of the PM may be impaired [7,8]. Biocompatibility of a PD solution can be defined as the capacity to leave the anatomical and functional characteristics unmodified in time. It can be divided into local (peritoneum cavity) and systemic. Now it is accepted that prolonged exposure to conventional PD fluids is harmful to the peritoneum, causing neoangiogenesis, inflammation, and fibrosis [9,10]. Damage to the PM is indicated by dwindling UF capacity eventually leading to UF failure, the main cause of PD failure [11].

Bioincompatibility in PD is attributed mainly to the high glucose (molecular weight 180 Da) load in the dialysate, the standard osmotic agent used in PD fluid due to its efficiency, low cost, and acceptable safety profile. Currently used PD solutions have a 10- to 50-fold higher glucose content than physiological serum levels; the osmotic gradient thus created makes it possible to remove water, electrolytes, and toxins by UF-associated convection [6]. The effects of such excess glucose, however, include not only a distinct role in the above-mentioned longitudinal changes to the peritoneal membrane but also many potential systemic metabolic effects, including insulin resistance, new-onset diabetes, and cardiovascular disease [12,13].

One of the key objectives of present-day research in PD is to devise strategies to reduce or eliminate glucose-associated toxicity (glucose sparing) without jeopardizing the patient’s health. However, finding effective and safe osmotic agents to be used in PD solutions has undoubtedly proved challenging. For PD clinical practice, only two alternative osmotic agents are currently available in glucose-free solutions: the glucose polymer icodextrin and amino acids. These formulations, either alone or in combination, have proved to be effective and PD patients may benefit from their use [14,15,16]. But icodextrin and amino acids replace no more than 30%–50% of the glucose absorbed every day [12], while they can only be used in a single daily peritoneal exchange [17,18]. Moreover, two recent randomized, controlled studies in PD patients showed that the combined use of icodextrin and amino acids improved metabolic indices, though some patients experienced extracellular fluid volume expansion [19]. The results of these studies emphasize the importance of efficacious UF and the need for close clinical monitoring of the patient’s fluid status with any glucose-sparing strategy. We also see from the data and experience published on commercially available glucose-sparing PD solutions (icodextrin, amino acids) that the future of PD depends largely on finding new osmotic agents improving its biocompatibility and the fluid balance, but also, and no less important, its effect on the metabolism.

The use of osmo-metabolic agents in the PD fluid represents a novel approach to antagonizing glucose-associated toxicity [20]. Osmo-metabolites are substances that have favorable osmotic and metabolic properties [21,22]. The osmo-metabolic approach would ensure a sort of bioactive glucose-sparing both by reducing intraperitoneal glucose load without compromising UF and by the independent mitigation of underlying systemic negative metabolic effects caused by the glucose load. L-carnitine and xylitol represent two such candidate agents. L-carnitine (molecular weight 161.2 Da) is highly water soluble and chemically stable in aqueous solutions [23], which renders it suitable for use in PD fluid. From our previous trials using carnitine-enriched PD solutions, we know the effectiveness of L-carnitine as an efficient osmolyte in PD [24], and that it also enhances CAPD patients’ insulin sensitivity [25]. Xylitol (molecular weight 151.2 Da), another osmo-metabolite, is a five-carbon sugar alcohol, pentitol, which is manufactured by the reduction of D-xylulose. A clinical trial many years ago [26] treated six insulin-dependent diabetic patients on CAPD for a minimum of five months using D-xylitol as the sole osmotic agent (three daily exchanges of PD solution with xylitol 1.5% and one exchange with xylitol 3%). Xylitol-containing PD fluid proved safe to use, maintained peritoneal UF, and significantly enhanced the patients’ glycemic control (the exogenous insulin dosage was halved, while glycosylated hemoglobin decreased significantly).

Osmo-metabolic agents can be used alone or in combination to maximize their therapeutic effects. We have recently developed a new PD solution containing L-carnitine, xylitol, and a low amount of glucose, and tested its effect on human vein endothelial cells obtained from the umbilical cords of healthy gestational diabetic mothers [22]. Such an experimental PD solution was not associated with the cytotoxicity, inflammation, or nitro-oxidative stress as found with a glucose-based, neutral pH, low-glucose degradation product PD solution, which is regarded as a “biocompatible” solution [27]. Moreover, very recently we compared this innovative PD solution formulation with a wide number of commercial PD solutions (including several “biocompatible” solutions), on human mesothelial cells cultured on inserts and only exposed to the PD solution on the apical side, which is what happens in a PD dwell [28]. The novel PD solutions showed improved performance in terms of cell viability, a better preserved integrity of the mesothelial layer, and less release of proinflammatory cytokines. Our studies also indicate that a little glucose can be retained in the PD fluid, in order to take advantage of its UF ability and energy-providing potential with patients who are often malnourished. Indeed, although the test solutions contained some glucose, it was at a lower concentration and did not seem to have the deleterious effects of the higher concentration [22,28].

Based on these results, the FIRST (efficacy and safety assessments of a peritoneal dialysis solution containing glucose, xylitol, and L-carnitine compared to standard PD solutions in CAPD) study was undertaken. Here we present the results obtained in the first cohort of patients completing the whole study period.

## 2. Results

### 2.1. Population Characteristics

Enrollment of eligible patients for the study was greatly hampered and delayed by the COVID pandemic and related implications. The study is currently ongoing. Reported here are the results obtained in the group of patients completing the study period at the Chieti center. Their main characteristics are shown in Table 1. Patients in group A were being treated with a 2.5% glucose dialysate for the nocturnal exchange. Patients in group B were being treated with a 1.5% glucose dialysate for two diurnal exchanges and with icodextrin dialysate for the nocturnal exchange.

During the four-week study period, patients included in group A received a bag with the experimental solution IPX15 for the nocturnal dwell; group B subjects received two bags with the experimental solution IPX07 for the daytime exchanges and a bag with icodextrin solution for the nocturnal dwell. The composition of the experimental bags is detailed in the Material and Methods section. Patients then returned to using their standard solutions in the four-week follow-up period.

### 2.2. Dialysis Efficiency Parameters

The following efficacy parameters were assessed during the study: total weekly urea Kt/V (a recognized index of dialysis adequacy in general), weekly total creatinine clearance (CrCL), peritoneal equilibration test (PET; a semiquantitative test to provide information about the transport characteristics of the peritoneal membrane), residual kidney function (RKF), daily diuresis, and daily peritoneal UF. The course of the parameters over the study period is shown in Table 2.

With regard to small solute clearance, in both groups of patients, Kt/V urea and creatinine clearance showed a slight increase at T28, thereafter declining toward baseline values (group A) or slightly increasing (group B). For residual kidney function, in both groups, a slight increase was found at T28 and a decrease at T56. Mean peritoneal UF in patients of group A proved to be increased at T28 and further increasing at T56, while in group B, peritoneal UF was quite stable. Daily urine volume had slightly increased in group A at both T28 and T56, whereas it proved to be decreased at both time points in group B. Evaluation of PM characteristics by PET showed that patients were average transporters. Small-solute transport, as expressed by D/P creatinine and D/D0 glucose, increased following intervention in both groups, whereas UF during PET showed a slight decrease in group A and a sustained increase in group B patients.

**Figure 1:**
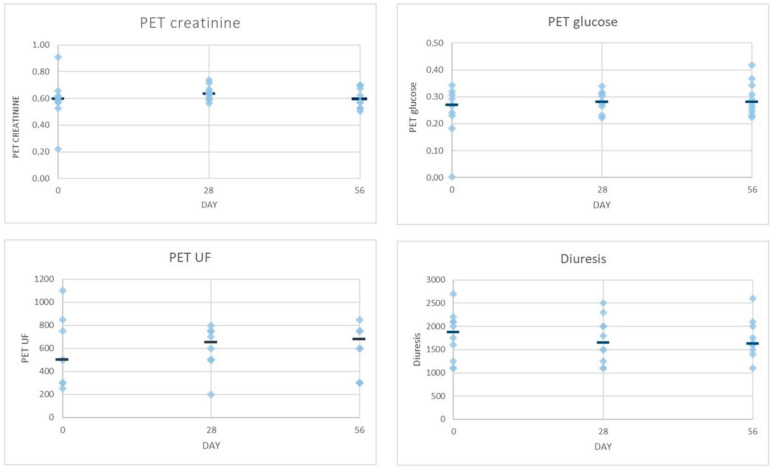
Individual data points and median (-) of peritoneal equilibration test (PET) and diuresis of group A and group B data lumped together. UF, ultrafiltration.

**Figure 2:**
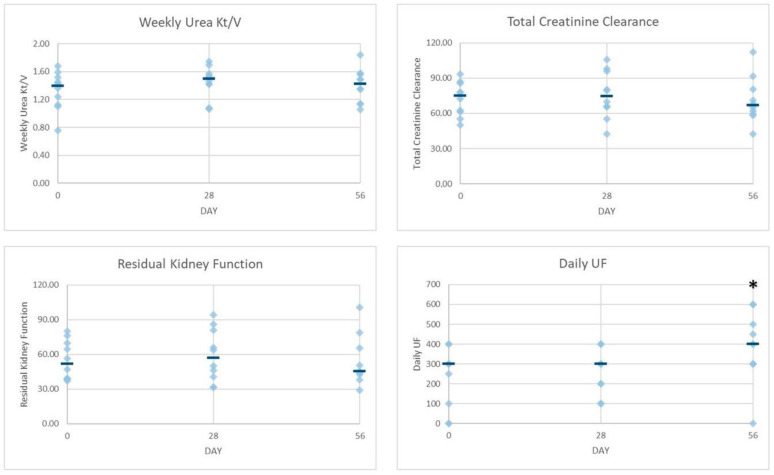
Individual data points and median (-) of dialysis efficiency parameters of group A and group B data lumped together. UF, ultrafiltration. * *p* < 0.02 vs. day 0.

In order to provide a graphical view of the various parameters at the different time points, Figure 1 and Figure 2 show individual data points together with the median of group A and B data lumped together. At first glance, in the overall picture with regard to the changes observed after the intervention and follow-up periods in small solute clearances, PM characteristics and UF were not so dissimilar than groups alone (Figure 1 and Figure 2). The smallest variability was observed in the PM characteristic at all time points, though a greater variability can be seen for the rest of the parameters evaluated, suggesting that more data are necessary to make any firm prediction. The sole significant difference when analyzing data at the three time points proved to be the daily peritoneal UF at T56 when compared to T0 (*p* < 0.02) (Figure 2).

### 2.3. Safety Results

There was good tolerance of the experimental PD solution, and no patient reported discomfort/pain during infusion. Vital signs, clinical examinations, and electrocardiographic findings did not raise safety concerns. No patient showed any serious signs of overhydration or had appreciable changes in body weight during the study. Medications did not change. The items of the subjective questionnaire on patient’s perception of well-being proved to be stable (the score was 18.8 ± 4, 18.3 ± 1.5, 16.5 ± 1.6 in group A and 16 ± 1, 16.5 ± 2.4, 17 ± 4 in group B, at T0, T28, and T56, respectively).

Biochemical parameters showed no significant changes at the different time points of the study (Table S1).

## 3. Discussion

Peritoneal dialysis is a feasible option for ESRD patients though it has remained underprescribed. This may be due to the high glucose load that current PD solutions cause the patient. The effects of such excess glucose include relatively early limitation of the UF capacity of the PM, and detrimental metabolic effects associated with intraperitoneal glucose absorption. Thus, great efforts are being made to develop alternative PD solutions avoiding these side effects; the aim is to replace part of the glucose content with other osmolytes no less efficient than glucose at removing fluids, but less damaging to the patient’s metabolism.

We have recently formulated PD solutions that replace part of the glucose load with other osmotically active metabolites, namely L-carnitine and xylitol [22,28]. This novel osmo-metabolic approach [20] gives the possibility of exploiting the pharmaco-metabolic properties of the two osmolytes to attenuate the systemic side effects due to glucose. Moreover, utilization of a novel PD solution replacing part of the glucose load with osmotically and metabolically active metabolites may give new insights into the potential positive impact of these novel osmo-metabolic agents on the convective phenomenon occurring in the PM during the continuous PD dwell time or throughout their absorption and effect on biologic pathways of solutes considered to be uremic toxins.

Here we have reported the preliminary results of the first clinical experience using osmo-metabolic agent-based PD solutions in CAPD patients. Use of L-carnitine and xylitol in the PD fluid over four consecutive weeks proved safe and well tolerated in all patients.

Urea Kt/V, creatinine clearance, PET-creatinine, PET-glucose, and RKF are representative of the efficacy of depuration/removal of small molecules through the peritoneal membrane and kidney, whereas diuresis, daily UF, and PET-UF indicate the efficacy of fluid removal through the peritoneal membrane and kidney. With regard to parameters of dialysis efficiency, both creatinine clearance and weekly urea Kt/V seemed to be slightly improved by the end of the intervention period in both group A and group B. Increasing removal of urea may benefit the uremic patient since a number of recent experimental data suggest that urea is toxic at concentrations representative for ESRD [29,30]. RKF seems to follow the same trend as the other dialysis efficacy parameters throughout the study. If further studies confirm that RKF may be longer preserved, uremic toxin removal will be an important advantage achieved by this novel PD solution. It should also be noted that these three parameters were moderately affected by variability of the data (Figure 2).

Interestingly, PM characteristics were much less affected by variability (Figure 1), whereas the PET creatinine seemed to follow the same trend as Kt/V, though PET glucose remained fairly stable throughout the study. If these data were confirmed, it might be speculated that our glucose-sparing solution improves the peritoneal clearance of small solutes without an increase in glucose absorption as expected by Twardowsky [31]. Daily UF did not differ throughout the study in group B, though, after the intervention period, group A showed a slight increase that became more marked by the end of the follow-up, suggesting a sort of carry-over effect. On the other hand, urine output remained fairly stable throughout the study in both experimental groups though with different trends (Table 2). In light of the glucose-sparing approach of our experimental PD solution, it should be noted that patients allocated in group A treated with one exchange of our experimental PD solution (IPX15) received only 20% (10 g vs. 50 g) of the daily glucose load when compared to the traditional glucose-based solution having the same osmotic strength, whereas patients allocated in group B treated with two exchanges of our experimental PD solution (IPX07) received only 30% (20 g vs. 60 g) of the daily glucose load when compared to patients treated with traditional glucose-based solution having the same osmotic strength.

Altogether, our data suggest the noninferiority of the novel solution compared to standard solution as far as adequacy and peritoneal transport characteristics are concerned. However, the results of the present report are clearly preliminary, having been obtained in a small-sized patient population treated for a short period of time, and this is certainly a limitation. The chief causes were difficulty in recruiting eligible patients and the extra intricacies of any controlled clinical trial involving outpatients during the COVID-19 pandemic. Notwithstanding this, the good tolerability and the encouraging data of this proof-of-concept study deserve further investigation in larger and longer studies. These studies are, respectively, ongoing (FIRST trial) or close to start (ELIXIR trial: a six-month randomized study to evaluate the efficacy and safety of XyloCore, a glucose-sparing experimental solution for PD), and will define the role of the proposed novel solutions in daily PD clinical practice.

## 4. Materials and Methods

### 4.1. Study Population

Stable patients with ESRD 18 years or older on CAPD therapy for at least three months were recruited in three Italian centers (Nephrology and Dialysis Unit of the University Hospital of Chieti, Bari, and Rome). Each patient gave written informed consent, and the study was approved by the local Ethics Committee of each center (project identification code IP-001-09; approved on 22/11/2018 by the Ethics Committee of G. d’Annunzio University of Chieti-Pescara, on 9/9/2020 by the Ethics Committee of Bari Policlinico Hospital, and on 5/11/2020 by the Ethics Committee of Rome Policlinico Gemelli). 

Prior to entering the study, patients needed to have been regularly treated by CAPD with a standard solution containing 2.5% glucose monohydrate (126.1 mmol/L, Dianeal; Baxter Healthcare, Mc Gaw Park, IL, USA) for the nocturnal dwell (group A), or with 1, 2, or 3 diurnal exchanges according to the patient’s need, using standard solutions containing 1.5% glucose monohydrate (75.5 mmol/L, Dianeal) combined with a nocturnal exchange with icodextrin (Extraneal; Baxter Healthcare, Mc Gaw Park, IL, USA) (group B).

Patients were required to be in stable clinical condition for four weeks before the screening period, as certified by medical/surgical history, physical examination, and laboratory exploration. Patients were excluded if they had received L-carnitine or its derivatives in the previous month or experienced a peritonitis episode in the previous three months. Other exclusion criteria included hemoglobin level <9 g/dL, severe diseases or acute infectious conditions, any history of major cardiovascular events like stroke, acute myocardial infarction, coronary, or other arterial revascularization procedures in the last three months before selection, pregnancy or lactation, or life expectancy less than 12 months.

### 4.2. Study Design

**Figure 3:**
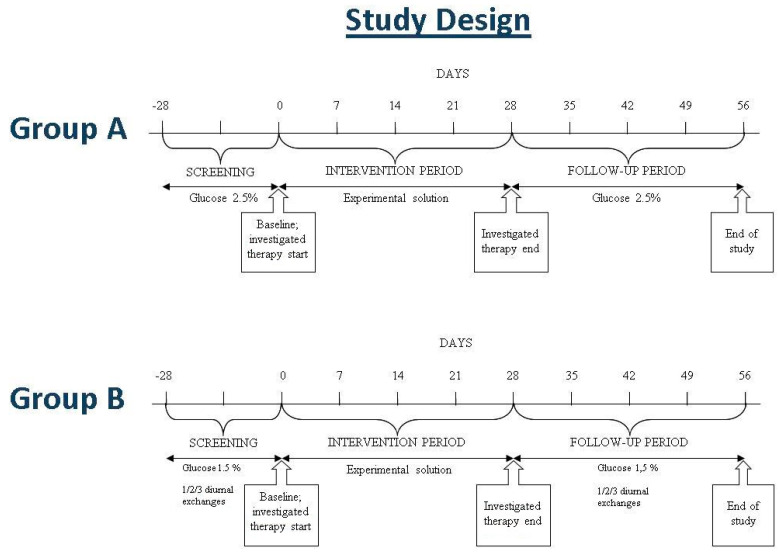
Study design.

FIRST is a phase II, prospective, investigational, open, multicenter study to investigate the tolerability and the efficacy of a new PD solution containing L-carnitine and xylitol in patients with ESRD receiving CAPD (NCT04001036). The study consists of three study periods (screening, intervention, and follow-up), with a total duration of around 84 days (Figure 3).

After a four-week run-in (screening period), dedicated to the identification of eligible subjects, enrolled patients entered the intervention period, which lasted four weeks. The subjects included in group A received a bag with experimental solution for the nocturnal dwell (IPX15). The subjects included in group B received 1, 2, or 3 bags with the experimental solution for the daytime exchanges (IPX07) and a bag with icodextrin solution for the nocturnal dwell. In the follow-up period of four weeks, patients returned to using standard solution with 2.5% glucose for the nocturnal exchange (group A) or to 1.5% glucose solution for diurnal exchanges and icodextrin for the nocturnal exchange (group B).

Target variables for safety and tolerability assessment of the experimental solutions included patient withdrawal from the study, incidence and severity of adverse events, concomitant medication, abnormal hematology and clinical chemistry measurements, clinical signs of overhydration, and changes in the subjective questionnaire on the patient’s perception of well-being.

Since the study has an explorative character, no primary and secondary efficacy parameters were identified. The following efficacy parameters were determined during the study: daily UF volume, weekly total urea Kt/V, weekly total creatinine clearance, and peritoneal equilibration test (PET). The 24 h urine volume was also measured.

### 4.3. Study Solutions

Study solutions were provided in sterile disposable 2 L bags (Galenica Senese, Monteroni D’Arbia, Siena, Italy). Bags had a pH of 5.5 and the following composition: sodium, 134 mmol/L; calcium, 1.75 mmol/L; magnesium, 0.5 mmol/L; chloride, 103.5 mmol/L; lactate, 35 mmol/L; glucose, 27.7 mM; and L-carnitine, 1.24 mM. Bags differed in their xylitol content: xylitol 98.6 mM (IPX15 solution) or xylitol 46 mM (IPX07 solution). The osmotic strength of our experimental PD solutions was comparable to the glucose-based PD solutions (see above) used before the intervention and follow-up periods. The experimental solutions used in this study were produced in accordance with Good Manufacturing Practice.

### 4.4. Study Procedures

**Figure 4:**
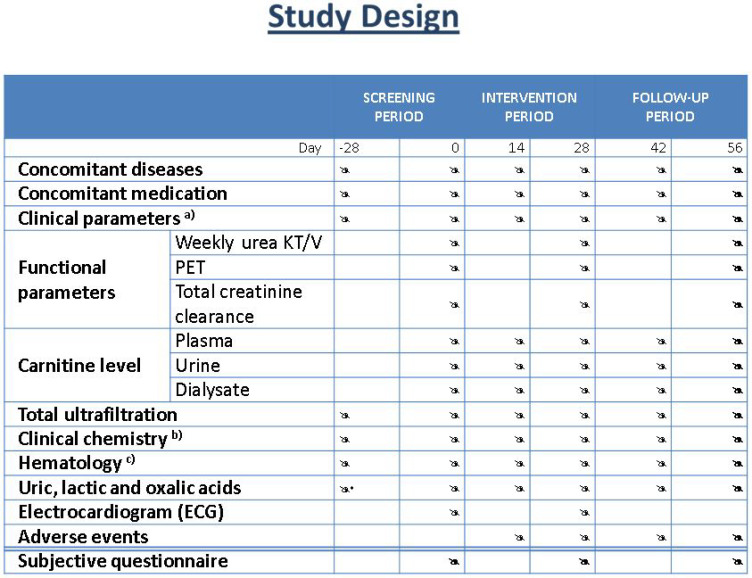
Study flowchart. **a**) Clinical parameters include diuresis. **b**) Clinical chemistry: serum sodium, potassium, calcium, phosphorus, total protein, albumin, GOT (AST), GPT (ALT), alkaline phosphatase, gamma-glutamyl transferase (GGT), total bilirubin, glucose, total cholesterol, triglycerides, HDL-cholesterol, LDL-cholesterol, blood urea nitrogen (BUN), and creatinine. **c**) Hematology consists of hemoglobin, hematocrit, red blood cell count, white blood cell count and differential, and platelet count. * at day -28, determination of uric acid only.

The study flowchart is reported in Figure 4.

Peritoneal UF was calculated in the following way: at each dwell, the fresh PD bag was weighed before and after flushing prior to the filling procedure, so as to correct for the flush-before-fill rinsing volume (no fixed volume being used) as well as for any over- or underfilling of the bag. From this last weight, we obtained the volume of infused PD solution by subtracting the weight of the empty bag. We measured the volume of the drained dialysate by weighing the drainage bag and again subtracting the empty bag weight. Peritoneal UF was calculated (mL) as drained (mL) − infused (mL) volume. Residual kidney function and parameters of dialysis adequacy including weekly urea Kt/V and creatinine clearance (defined as residual renal clearance + dialysate clearance) were determined as detailed in Appendix A.

A standard PET was used to assess PM transport characteristics. It consisted of a 4 h dwell with 3.86% glucose during which period we collected dialysate samples at times 0, 120, and 240 min, while a blood sample was taken at 240 min. All blood and dialysate samples were then analyzed within 24 h. The dialysate’s creatinine concentration was corrected for interference with glucose in the effluent. The D/P creatinine was calculated as the ratio of dialysate creatinine concentration at 240 min with respect to serum concentration; the D/D0 glucose was obtained as the ratio of dialysate glucose concentration at 240 min to time 0; while the UF volume was gauged from the difference between the 4 h drain and instillation volumes.

All measurements were performed in a fasting state. Blood samples obtained for hematology, clinical chemistry, and uric and lactic acids were analyzed by standard laboratory techniques. Plasma oxalate was enzymatically determined according to Ladwig et al. [32]. Free L-carnitine and acyl-carnitine esters will be measured in plasma, urine, and peritoneal solution drained out by high-performance liquid chromatography/mass spectrometry [33]. Carnitine measurements are not available yet as, according to the clinical protocol, carnitines determinations will be conducted in a centralized laboratory at the end of the clinical trial.

A subjective questionnaire on patient perception of well-being was also administered. The questionnaire was completed at T0, T28, and T56 and included 15 items: nausea, asthenia, lack of appetite, constipation, diarrhea, stomach pain, muscle aches, muscle cramps, itching, breathing difficulties, chest pain, fatigue, feeling faint, tingling in the hands and feet, problems with the peritoneal catheter. Each item was given a score that ranged from 0 to 5, based on the intensity of the symptom: score 0 corresponded to slight intensity, score 5 to severe intensity. The higher the global score, the worse the perception of well-being.

### 4.5. Data Analyses

Because of the lack of available data regarding the effects of studied pharmacological association in ESRD patients undergoing CAPD, calculation of sample size was done on the basis of a subjective questionnaire as to the patient’s perception of well-being. Forty patients with ESRD treated by CAPD will be included, 20 in group A and 20 in group B.

But for Table 1, data are reported as median (interquartile range). Due to the smallness of the sample (group A, *n* = 6; group B, *n* = 4), the statistical analysis operated with median and interquartile range with the adding of nonparametric repeated measures test (Friedman test) and, if the previous test was significant at the 0.05 level, post hoc Wilcoxon test for the comparison among time points. Scatterplots were used to visualize the distribution of values at the three time points by lumping the data of group A and B evaluated for each parameter in the clinical trial. SAS VERSION 9.4 package (SAS Institute corp., Cary, NC, USA) was used to conduct statistical analyses.

## Figures and Tables

**Table 1:** Characteristics of study population.

	Group A	Group B
Number of patients	6	4
Age (years)	69.8 ± 5.2	55.7 ± 1 2.4
Gender (male/female)	3/3	4/0
Body mass index (kg/m^2^)	28.8 ± 5.6	28.3 ± 1.2
Systolic blood pressure (mm Hg)	134 ± 22	135 ± 17
Diastolic blood pressure (mm Hg)	82 ± 8	81 ± 10
Heart rate (beats/min)	65 ± 10	77 ± 15
Time on dialysis (months)	6.7 ± 2.6	6.3 ± 0.5

Data are expressed as number or mean ± standard deviation.

**Table 2:** Parameters of dialysis efficiency during the study period.

Group A			
	Day 0	Day 28	Day 56
Urea Kt/V (weekly)	1.34 (1.12–1.52)	1.42 (1.08–1.57)	1.32 (1.14–1.49)
Net peritoneal UF (mL/day)	175 (0–300)	200 (100–300)	350 (300–500)
Residual kidney function (L/week) *	60.5 (39.0–76.2)	64.8 (46.3–85.9)	48.0 (42.8–78.8)
Creatinine clearance (L/week) *	77.9 (55.5–85.5)	79.9 (55.5–95.8)	62.5 (58.0–91.5)
Solute transport (D/P creatinine)	0.59 (0.57–0.62)	0.62 (0.60–0.66)	0.57 (0.53–0.62)
Solute transport (D/D0 glucose)	0.26 (0.23–0.31)	0.28 (0.27–0.28)	0.30 (0.27–0.37)
Urine output (mL/day)	1425 (1100–2000)	1500 (1100–2000)	1550 (1400–1750)
**Group B**			
	Day 0	Day 28	Day 56
Urea Kt/V (weekly)	1.41 (1.06–1.52)	1.53 (1.49–1.64)	1.53 (1.49–1.64)
Net peritoneal UF (mL/day)	350 (300–400)	350 (300–400)	400 (350–425)
Residual kidney function (L/week) *	43.0 (38.3–58.3)	45.2 (36.0–65.4)	44.7 (42.1–55.4)
Creatinine clearance (L/week) *	67.4 (62.0–79.6)	67.9 (65.7–84.0)	69.6 (65.3–75.8)
Solute transport (D/P creatinine)	0.60 (0.56–0.64)	0.65 (0.60–0.70)	0.69 (0.60–0.70)
Solute transport (D/D0 glucose)	0.28 (0.23–0.32)	0.31 (0.27–0.33)	0.25 (0.24–0.30)
Urine output (mL/day)	2100 (1925–2150)	1900 (1525–2250)	1825 (1625–2050)

Data are expressed as median (interquartile range). Day 0–day 28, use of the experimental PD solution; day 28–56, use of standard solution. * normalized to body surface area. Abbreviations and definitions: net peritoneal UF, difference between total peritoneal drained volume and total peritoneal filling volume; UF, ultrafiltration; D/P creatinine, dialysate to plasma creatinine ratio during the standard peritoneal equilibration test; D/D0, dialysate glucose concentration ratio between the end and beginning of peritoneal equilibration test.

## Data Availability

The data that support the findings of this study are available from Iperboreal Pharma (Italy), but restrictions apply to the availability of these data, which were used under license for the current study, and so are not publicly available. Data are, however, available from the authors upon reasonable request and with permission of Iperboreal Pharma (Italy).

## Supplementary Materials

The following are available online at https://www.mdpi.com/2072-6651/13/3/174/s1, Table S1. Main laboratory parameters.

## Appendix A

Formulas used to calculate residual kidney function and dialysis efficiency parameters

*Residual Kidney Function*

[(urinary creatinine concentration/serum creatinine concentration) × urinary volume × 7) + (urinary urea concentration/serum urea concentration) × urinary volume × 7]/2

The value obtained was then normalized to the body surface area (BSA):

(residual kidney function × 1.73 m^2^ BSA)/patient BSA

BSA **=** 0.007184 × body weight (kg)^0.425^ × Height (cm)^0.725^ (DuBois formula)

*Total weekly creatinine clearance = residual renal creatinine clearance + dialysate creatinine clearance*

[(Residual renal creatinine clearance + residual renal urea clearance)/2] + dialysate creatinine clearance

Residual renal creatinine clearance = (urinary creatinine concentration/serum creatinine concentration) × urinary volume × 7

Residual renal urea clearance = (urinary urea concentration/serum urea concentration) × urinary volume × 7

Dialysate creatinine clearance = (dialysate creatinine concentration × serum creatinine concentration) × dialysate volume × 7

The value obtained was then normalized to the body surface area:

Normalized total weekly creatinine clearance = creatinine clearance × 1.73 $m^{2}$BSA)/patient BSA

*Total weekly urea Kt/V = residual renal Kt/V+ dialysate Kt/V*

Residual renal Kt/V: [((urine urea concentration/serum urea concentration) × (urinary volume × 1000/1440)) × 1440 × 7] × body weight (kg) × 0.6 (0.55 if female) × 1000

Dialysate Kt/V: ((dialysate urea concentration/serum urea concentration) × (dialysate volume × 7)) × body weight (kg) × 0.6 (0.55 if female)
