# Supplementary material for: A New Peritoneal Dialysis Solution Containing L-Carnitine and Xylitol for Patients on Continuous Ambulatory Peritoneal Dialysis: First Clinical Experience
Source: Toxins (Basel). 2021 Feb 24;13(3):174. doi: 10.3390/toxins13030174 (PMC7996173; doi:10.3390/toxins13030174)
Supplement: Supplementary file 1 [file toxins-13-00174-s001.pdf]

Supplementary Materials: A New Peritoneal Dialysis Solution Containing L-Carnitine and Xylitol for Patients on Continuous Ambulatory Peritoneal Dialysis: First Clinical Experience

Carmela Rago, Teresa Lombardi, Giorgia Di Fulvio, Lorenzo Di Liberato, Arduino Arduini, José C. Divino-Filho and Mario Bonomini

Table 1. Main Laboratory parameters during the study period.

GROUP A			
	Day 0	*Day 28*	*Day 56*
**Urea (mg/dL)**	163.5 (113.0 - 202.0)	178.0 (112.0 – 245.0)	177.5 (127.0 – 192.0)
**Creatinine (mg/dL)**	7.7 (5.6 – 10.0)	7.2 (5.4 – 10.0)	7.1 (5.7 – 11.2)
**Total Cholesterol (mg/dL)**	173.0 (143 – 196)	170.0 (136.0 – 245.0)	172.5 (134.0 – 236.0)
**Cholesterol HDL (mg/dL)**	34.5 (25.0 – 52.0)	34.0 (27.0 – 56.0)	35.0 (26.0 – 48.0)
**Cholesterol LDL (mg/dL)**	100.5 (70.0 – 114.0)	91.0 (63.0 – 133.0)	96.0 (67.0 – 141.0)
**Triglyceride (mg/dL)**	131.0 (85.0 – 364.0)	154.0 (107.0 – 344.0)	164.5 (122.0 – 250.0)
**Haemoglobin (g/dL)**	11.1 (8.5 – 13.2)	10.9 (10.1 – 12.2)	10.8 (8.2 – 12.3)
**White Cell Count (×10^3/uL)**	6.3 (4.7 – 14.3)	7.0 (4.9 – 10.6)	6.7 (5.2 – 8.0)
**Platelet (×10^3/mmc)**	225.5 (187.0 – 301.0)	240.5 (226.0 – 344.0)	242.5 (214.0 – 314.0)
**Uric Acid (mg/dL)**	5.2 (4.3 – 9.9)	5.3 (4.1 – 6.9)	5.6 (5.2 – 7.3)
**Lactic Acid (mg/dL)**	9.5 (7.0 – 21.0)	10.5 (7.0 – 13.5)	9.0 (6.0 – 20.0)
**Oxalic Acid (umol/L)**	72.8 (62.2 – 198.2)	91.2 (37.0 – 167.1)	83.3 (51.8 – 113.0)
**GROUP B**			
	Day 0	*Day 28*	*Day 56*
**Urea (mg/dL)**	185.9 (169.0 – 219.0)	182.5 (174.0 – 212.0)	164.0 (154.0 – 174.0)
**Creatinine (mg/dL)**	9.3 (7.0 – 12.8)	9.6 (7.2 – 12.7)	9.9 (8.3 – 11.8)
**Total Cholesterol (mg/dL)**	133.5 (113.0 – 149.0)	134.0 (123.0 – 142.0)	125.0 (120.0 – 158.0)
**Cholesterol HDL (mg/dL)**	40.5 (25.0 – 49.0)	42.5 (31.0 – 47.0)	35.0 (33.0 – 38.0)
**Cholesterol LDL (mg/dL)**	72.5 (56.0 – 75.0)	69.0 (61.0 – 79.0)	70.0 (60.0 – 83.0)
**Triglyceride (mg/dL)**	122.5 (77.0 – 189.0)	101.0 (97.0 – 179.0)	100.5 (74.0 – 171.0)
**Haemoglobin (g/dL)**	11.4 (8.9 – 11.8)	10.7 (8.6 – 12.3)	10.6 (10.1 – 11.9)
**White Cell Count (x10^3/uL)**	8.2 (6.1 – 31.2)	8.6 (6.5 – 35.8)	7.3 (7.0 – 29.9)
**Platelet (x10^3/mmc)**	183.5 (132.0 – 263.0)	199.5 (134.0 – 305.0)	171.5 (133.0 – 399.0)
**Uric Acid (mg/dL)**	4.2 (3.3 – 5.2)	6.6 (4.3 – 7.7)	5.3 (3.9 – 6.8)
**Lactic Acid (mg/dL)**	8.5 (6.0 – 15.3)	9.9 (8.1 – 17.1)	11.0 (6.0 – 17.7)
**Oxalic Acid (umol/L)**	57.4 (37.9 – 110.9)	79.1 (71.1 – 116.8)	30.0 (14.4 – 57.3)

Results are expressed as median (interquartile range).
